# Linkages between rumen microbiome, host, and environment in yaks, and their implications for understanding animal production and management

**DOI:** 10.3389/fmicb.2024.1301258

**Published:** 2024-01-29

**Authors:** Weiwei Wang, Yuntao Dong, Wei Guo, Xiao Zhang, A. Allan Degen, Sisi Bi, Luming Ding, Xiang Chen, Ruijun Long

**Affiliations:** ^1^Laboratory of Animal Genetics, Breeding and Reproduction in the Plateau Mountainous Region, Ministry of Education, College of Animal Science, Guizhou University, Guiyang, Guizhou, China; ^2^State Key Laboratory of Grassland Agro-Ecosystems, College of Ecology, Lanzhou University, Lanzhou, China; ^3^Tianjin Key Laboratory of Conservation and Utilization of Animal Diversity, College of Life Sciences, Tianjin Normal University, Tianjin, China; ^4^Desert Animal Adaptations and Husbandry, Wyler Department of Dryland Agriculture, Blaustein Institutes for Desert Research, Ben-Gurion University of the Negev, Beer Sheva, Israel

**Keywords:** environmental adaptation, host metabolic regulations, rumen microbiome, host-rumen microbiome-environment linkages, management implications

## Abstract

Livestock on the Qinghai-Tibetan Plateau is of great importance for the livelihood of the local inhabitants and the ecosystem of the plateau. The natural, harsh environment has shaped the adaptations of local livestock while providing them with requisite eco-services. Over time, unique genes and metabolic mechanisms (nitrogen and energy) have evolved which enabled the yaks to adapt morphologically and physiologically to the Qinghai-Tibetan Plateau. The rumen microbiota has also co-evolved with the host and contributed to the host's adaptation to the environment. Understanding the complex linkages between the rumen microbiota, the host, and the environment is essential to optimizing the rumen function to meet the growing demands for animal products while minimizing the environmental impact of ruminant production. However, little is known about the mechanisms of host-rumen microbiome-environment linkages and how they ultimately benefit the animal in adapting to the environment. In this review, we pieced together the yak's adaptation to the Qinghai-Tibetan Plateau ecosystem by summarizing the natural selection and nutritional features of yaks and integrating the key aspects of its rumen microbiome with the host metabolic efficiency and homeostasis. We found that this homeostasis results in higher feed digestibility, higher rumen microbial protein production, higher short-chain fatty acid (SCFA) concentrations, and lower methane emissions in yaks when compared with other low-altitude ruminants. The rumen microbiome forms a multi-synergistic relationship among the rumen microbiota services, their communities, genes, and enzymes. The rumen microbial proteins and SCFAs act as precursors that directly impact the milk composition or adipose accumulation, improving the milk or meat quality, resulting in a higher protein and fat content in yak milk and a higher percentage of protein and abundant fatty acids in yak meat when compared to dairy cow or cattle. The hierarchical interactions between the climate, forage, rumen microorganisms, and host genes have reshaped the animal's survival and performance. In this review, an integrating and interactive understanding of the host-rumen microbiome environment was established. The understanding of these concepts is valuable for agriculture and our environment. It also contributes to a better understanding of microbial ecology and evolution in anaerobic ecosystems and the host-environment linkages to improve animal production.

## 1 Introduction

Ruminants play an essential role in global human societies due to their unique ability, via their rumen microbiome, to convert low-quality feedstuffs into valuable animal products such as milk and meat for human consumption. The rumen microbiota exerts a profound influence on dietary nutrient metabolism, the quality of animal products, animal production, and the environment (Mizrahi et al., 2021). Over the past decades, the rumen microbiome has emerged as central to tackling major challenges associated with the global demand for agriculture, rising animal protein demands (milk and meat products, 63% and 76%, respectively) (Alexandratos and Bruinsma, 2012; Huws et al., 2018), approximately 18% of total methane (CH_4_) emissions from all anthropogenic sources (Mizrahi et al., 2021), and sustainable and efficient ruminant production along with land constraints (Huws et al., 2018).

Ruminant performance is not only affected by host genetics but also by the environment and the microorganisms that inhabit the rumen (Brito et al., 2020; Mizrahi et al., 2021). Genetics and environmental determinants and their interactions have guided empirical and theoretical research in animal production and ecology for decades (Brinks et al., 1962; Angilletta and Sears, 2011). Studies have reported that rumen microorganisms can provide more than 70% of the host's metabolic and protein requirements (Siciliano-Jones and Murphy, 1989; Bergman, 1990). The integrated interactions between the host, rumen microbiome, and the environment, therefore, mutually contribute to animal performance. Understanding the complex linkages between the rumen microbiota, the host, and the environment is essential to optimize rumen function to meet the growing demands for animal products while concurrently minimizing the environmental impact of ruminant production (Huws et al., 2018; Mizrahi et al., 2021). However, to date, little is known about the mechanisms related to host-rumen microbiome-environment linkages and how they ultimately benefit the animal in adapting to changes to optimize their performance.

More than 14 million yaks (*Bos grunniens*) are raised on the Qinghai-Tibetan Plateau. They are essential for the livelihood of the local inhabitants as they provide meat, milk, dung, fiber, and transport (Long et al., 1999a). Under the harsh environmental conditions, yaks have evolved and adapted themselves morphologically (Shao et al., 2010), physiologically (Ishizaki et al., 2005), and genetically (Qiu et al., 2012) to the severe Qinghai-Tibetan Plateau (QTP). Yaks are more efficient in utilizing the poor-quality, high-fiber forage that is mainly available for long periods on the QTP. Previous studies have reported that the rumen microbiota in yaks enables the host to survive the extreme environment (Zhang Z. et al., 2016; Mizrahi et al., 2021). In this review, we piece together the yak's unique adaptation to the QTP ecosystem by describing the rumen microbiome of yaks and the obligatory dependence of yaks on their microbes for the degradation and digestion of the plants they ingest. The linkages between host genes, metabolisms, and rumen microorganisms that coordinate and affect the quality of yak milk and meat are clarified. The review offers a systemic, integrated perspective on the host-rumen microbiome-environment linkages while attempting to decipher their key interactions for the purpose of understanding and regulating animal performance as a whole.

## 2 The harsh environment shapes the unique nutritional deprivation adaptation of yak

**Figure 1:**
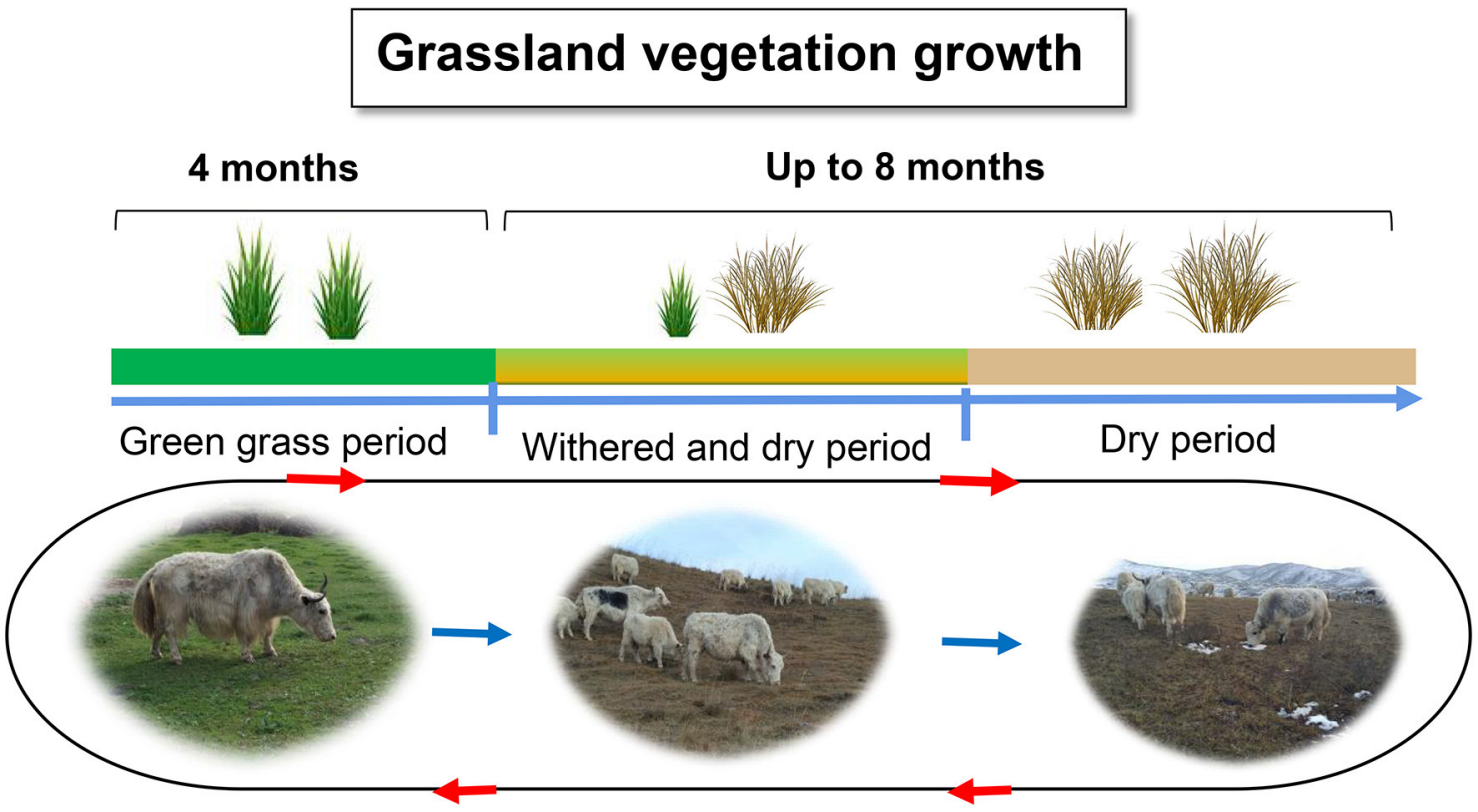
Yaks exposed to natural pastures of different qualities.

The Qinghai-Tibetan Plateau, regarded as the “Third Pole” and commonly referred to as the “Roof of the World,” has an average elevation exceeding 4,000 m above sea level (m a.s.l.). The high-altitude Qinghai-Tibetan Plateau is characterized by severe cold, low atmospheric pressure, oxygen partial pressure, and intense ultraviolet light, resulting in a short forage growing season (Long et al., 2008). Winters are particularly severe for grazing livestock, as the availability of forage is frequently insufficient and of subpar quality. The cold season could last for 8 months, especially in the dry forage phase in autumn and winter, during which time the cellulose and lignin contents gradually increase while the crude protein content decreases (Figure 1) (Long et al., 1999a). Neutral detergent fiber (NDF) contents account for up to 65.7% and crude protein account for only 2.96%−6.81% of the pasture dry matter from November to April (Xie and Chai, 1996; Ding et al., 2014). This nutritional shortage could result in a body weight loss of 30% or could even cause the death of the livestock (Long et al., 1999b). Such harsh environmental conditions have shaped the yaks' extraordinary nutritional deprivation adaptations to the harsh QTP. Yaks have demonstrated distinct energy and protein metabolic adaptations and host gene regulation.

### 2.1 Energy metabolism

Basal energy metabolism is defined as the minimal level of heat production during complete rest in a thermoneutral environment. It was estimated that the maintenance energy requirement for yaks is 458 kJ/kg BW^0.75^ (Han and Xie, 1991), and heat production scales to BW^0.52^ rather than BW^0.75^ (Hu, 1994; Hu et al., 2002). Diverging from other mammals, yaks exhibit a reduced metabolic rate as air temperature decreases, thereby employing a unique way of energy conservation (Han et al., 2002; Ding et al., 2014). At the lowest of the three elevations in the trials conducted by Hu (2001), the absolute fasting heat production of yaks exceeded that of yellow cattle (*Bos taurus*). However, such a distinction was not evident at the higher altitudes in their study. At 1–3 years of age, fasting heat production per day of yak at the altitudes of 3,250 m and 4,271 m were 329 kJ/kgW^0.75^ and 281–376 kJ/kgW^0.75^, respectively, compared to yellow cattle at 353–414 kJ/kgW^0.75^ and 360–516 kJ/kgW^0.75^, respectively (Hu and Xie, 1992; Han et al., 2002).

### 2.2 Nitrogen metabolism

Several studies have reported a higher efficiency of utilization of dietary nitrogen (N) in yaks than in lower-altitude cattle (Long et al., 1999c; Xue and Han, 2001; Wang et al., 2011). A few other studies reported the low N requirements for maintenance [0.40–0.53 g/(kg W^0.75^·d)] by yaks (Hu, 2001; Long et al., 2004). For instance, Guo et al. (2012) found that 87% of the urea synthesized in the liver of yak could be recycled into the digestive tract, providing nitrogen for rumen microbes to synthesize microbial proteins. With low N intake, rumen microbes are almost the only source of digestible protein for the host (Ørskov, 1982). Under such conditions, yaks utilized urea-N to meet the requirements of nitrogen for nearly 6 months (Wang et al., 2002), which could well point to an adaptive response of the yak to life at high altitudes and to the nutritional deprivation that yaks experience in winter and early spring.

### 2.3 Genes' regulation

Gene families related to nutrition assimilation and utilization and energy metabolism have expanded dramatically in the yak genome (Qiu et al., 2012). Qiu et al. (2012) linked nutrition metabolism from the field of yak host genes in comparison with cattle. These authors' study revealed five genes that were involved in integrated nutrition pathways and positively selected in the yak lineage. Among the genes, *HSD17B12, GLUL, GCNT3*, and *WHSCL* play important roles in the metabolism of fatty acids, amino acids, and polysaccharides. *GLUL* may be vital for the high level of nitrogen utilization in yaks (Qiu et al., 2012). Correspondingly, in nitrogen cycling, the PepT1 expression was found to be enriched in the yak epithelium of the small intestine compared to cattle epithelium (Wang et al., 2016). The positively selected changes in *CAMK2B* play a regulatory role in the secretion of gastric acid in the rumen, thereby contributing to the assimilation of short-chain fatty acids (SCFAs) that provide 70% of metabolic energy for the host and are produced by ruminal fermentation (Bergman, 1990; Qiu et al., 2012). Through the transcriptome analysis of rumen wall epithelial cells in yak and cattle, it was further revealed that 36 genes associated with the energy (SCFAs) and translocation were upregulated in yak compared to cattle. The genes were the following: SCFA transport: *PLA2G5, FABP3, CLCN1, GABRA3, BEST1, SLC12A3, SLC4A11, P2RY4, P2RY6, SLC4A7, SLC20A2, SLC13A3, SLC4A3, SLC13A5, SLC6A6*, and *SLC16A6*; fatty acid metabolic process: *ALOX5AP, SYK, ABCD1, CPT1C, PLA2G15, PTGES, LTC4S, PRKAA1, PRKAB2, BRCA1*, and *MCAT*; regulation of carbohydrate metabolic process: *IFNG, DYRK2*, and *SPDYA*; Glycolysis: *LDHC, PKLR*, and *HK3*; organic acid catabolic process: *DDO*; and pyruvate metabolic process: *PC* and *ENO2* (Zhang Z. et al., 2016).

## 3 Rumen microbiome services are synergetic linkages with nutritional deprivation

The host functions as an ecosystem within which microbial processes play out. The gut microbiota of importance in ruminants maintains a healthy state for the host, especially the rumen microbiota. The biomass of rumen microorganisms is comprised of protozoa (~50%), fungi (~8%), bacteria (largely distributed), archaea (0.3%−4%), and little proportion of as yet largely uncharacterized viruses, especially phages (Tapio et al., 2017; Gilbert et al., 2020; Lobo and Faciola, 2021). In comparison with cattle, rumen microbial activity provides the following services for yaks:

### 3.1 Lower methane emission

**Figure 2:**
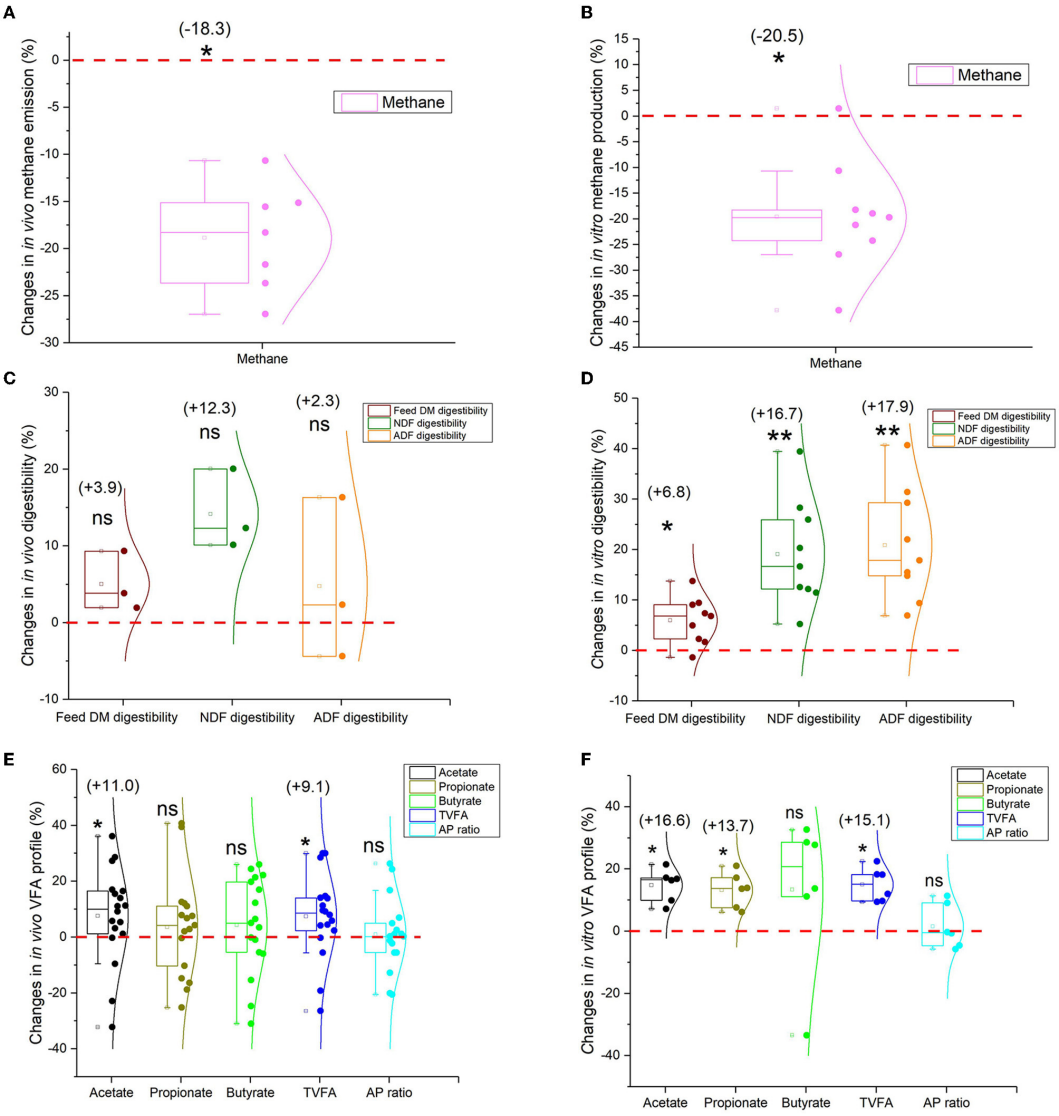
Box and whisker plots of rumen fermentation profiles comparing yaks and cattle. **(A)** The *in vivo* methane emission comparison (*n* = 7). **(B)** The *in vitro* methane production comparison (*n* = 9); **(C)** The *in vivo* comparison of feed digestibility (*n* = 3); **(D)** The *in vitro* comparison of feed digestibility (*n* = 9); **(E)** The rumen fermentation VFA profile (*in vivo*) (Acetate and TVFA: *n* = 17; propionate, butyrate, and AP ratio: *n* = 16); **(F)** The *in vitro* rumen fermentation VFA profile (*n* = 6) (see Supplementary Tables 1–5). Values in parentheses indicate the percentage from the statistical analysis comparing yaks with cattle (cattle represent baseline as zero). Wilcoxon Signed Rank test: **p < 0.01; *p < 0.05; ns = not significantly different from zero. TVFA, total volatile fatty acid; DM, dry matter; NDF, neutral detergent fiber; ADF, acid detergent fiber; AP ratio, acetate to propionate molar ratio.

Methane (CH_4_), a byproduct from ruminal fermentation by methanogens, results in energy loss, and the energy equivalent amounts to 2%−15% of gross energy intake (Moss et al., 2000; Shabat et al., 2016). Annual CH_4_ emissions from ruminants are approximately 2.1 GtCO2e (gigatons of carbon dioxide equivalent), accounting for 20.2% of global CH_4_ emissions (IPCC, 2019). Methane emission of yaks is less than other ruminants and has been confirmed through various approaches (mask, SF_6_, model estimation, and *in vitro* gas technique) (Figures 2A, B, Supplementary Tables 1, 2), indicating that yaks are low-carbon emission animals and are proven to have high efficiency (Shabat et al., 2016). The rumen microbiome determines the amount of CH_4_ production.

Studies of bacteria reported that S24-7, *Butyrivibrio, Shwartzia, Treponema, Clostridium*, RFP12, *Coriobacteriaceae*, and *Methanosphaera* reduce CH_4_ emissions and improve animal production performance (Cunha et al., 2017). Under grazing conditions, the core genus (relative abundance >0.5%) of the yak is YRC22, with unidentifiable BS11 and BF311 and unidentified S24-7 and CF231 in *Bacteroidetes*; *Treponema* in *Spirochaetes*; *Clostridium sensustricto* in *Firmicutes*; and unrecognized RFP12 in *Verrucomicrobia*. *Treponema* and RFP12 are enriched in the grazing yak (Xue et al., 2017), which might be associated with the lower enteric CH_4_ emission.

It has been reported that the percentage of archaea in the rumen microorganisms is only approximately 0.3%−4%, but the percentage is vital as hydrogen sinks in the rumen and for CH_4_ emissions from ruminants. It is speculated that the low-methane emission trait may be due to the high diversity of archaea; the archaeal diversity in grazing yaks is higher than that in yellow cattle (Huang et al., 2016), which was also reported in another study (Xue et al., 2016). A high diversity in low CH_4_ emitters has been confirmed by Auffret et al. (2017). *Methanobrevibacter* (>60%), *Methanomicrobium* (~15%), and *Methanomassiliicoccales* (~16%) are the abundant genera in the archaeal community (St-Pierre and Wright, 2012; Borrel et al., 2014), while *Methanobacteriales* and *Thermgymnomonas* have been reported as the dominant archaea in yaks (Huang et al., 2012, 2016; Mi, 2016; Wang et al., 2017). *Thermogymnomonas*, which belongs to Thermoplasmatales-affiliated Lineage C (TALC), has the highest abundance. *Methanobrevibacter*, composing only 25% (Wang et al., 2017), and a large number of unknown methanogen TALCs were found in yaks (Huang et al., 2012). *Thermoplasmatales*, belonging to the family *Methanomassiliicocaceae* (Rumen cluster C, RCC), is a major component of methanogens (Janssen and Kirs, 2008; Poulsen et al., 2013; Borrel et al., 2014) with a methylotrophic methanogenesis pathway; methylotrophic methanogens, belonging to the class Thermoplasmata, have been associated with decreased CH_4_ production (Poulsen et al., 2013); and relative abundances are significantly higher in yaks than in cattle (*Bos taurus*) (Zhang Z. et al., 2016). Although the majority of methane is generated via the hydrogenotrophic methanogenesis pathway, i.e., utilizing H_2_ and CO_2_ or formate as substrates rather than acetate, the methylotrophic methanogenesis pathway, which employs methanol and methylamines as substrates, also contributes to a certain extent (Carberry et al., 2013), indicating that the methanogenesis pathway is unusual in yaks. *Methanomassiliicoccales* could also provide natural protection in the gut (Brugère et al., 2014) and tend to be more abundant in high-feed-efficiency ruminants (Li et al., 2016; Li and Guan, 2017). *Methanobrevibacter gottschalkii*, an archaea of higher abundance in cattle than yak (Zhang Z. et al., 2016), has also been correlated positively with CH_4_ emission (Tapio et al., 2017).

The density of protozoa in the rumen is between 10^4^ and 10^6^/ml, and its biomass is large, accounting for more than half of the rumen microorganisms. The total number of ciliates ranges from 0.7 to 8.5 × 10^5^/ml in yaks (Bi et al., 1989; Xie et al., 1989; Gui et al., 2000; Yao et al., 2002), which is lower than that in cattle (*Bos taurus*) and buffaloes (*Bubalus bubalis*) (Ito et al., 1994; Chaudhary et al., 2000). Rumens with fewer protozoa possess fewer methanogens, which may be one of the reasons for low CH_4_ emissions in yaks. The functional genes of the protozoa, such as mcr A and fmd B genes, are also associated with CH_4_ emissions (Roehe et al., 2016). The role of the protozoa has been reported as contributing to the maintenance of prokaryotic diversity in the rumen and potentially mitigating the impact of competitive exclusion among bacterial taxa (Solomon et al., 2022). However, the role of protozoa in yaks during methane production needs further research.

Sequencing of the rumen microbiome demonstrated that microbial genes are directly associated with CH_4_ emissions. In yellow cattle, the gene enrichment included the CO_2_/H_2_ and methanogenic pathways (Zhang Z. et al., 2016), indicating the higher energy efficiency of yak ruminal microbes in utilizing crude feed. Similarly, in low-efficient dairy cows, the methanogenic metabolic pathway is enriched, while in the high-efficiency groups, the lactic acid-propionate conversion pathway is enriched (Shabat et al., 2016). In rumen anaerobic fermentation, stoichiometric laws of chemical balance are maintained between the amount of metabolic hydrogen released during carbohydrate oxidation and the amount of hydrogen incorporated into the reduced end products, namely, methane, propionate, and butyrate (Immig, 1996). Yaks have more unknown hydrogen sinks and fewer hydrogen sinks shifting to methanogenesis than other ruminants (Wang, 2020). An increase in the ratio of yak to cattle rumen inoculum decreased CH_4_ production and increased fiber digestion and VFA profile *in vitro*. The reduced CH_4_ production, possibly attributed to reductive acetogenesis competing for CO_2_ and H_2_ as intermediate, aligns with the decrease in metabolic hydrogen recovery (*[2H]*_*recovery*_) as the yak rumen inoculum increases, which indicates that reductive acetogenesis may elucidate a portion of the unexplained metabolic hydrogen ([2H]) in the fermentation of the yak rumen inoculum. Reductive acetogenesis herein might be the case for yaks exerting more function in their digestive tract than other bovines (Joblin, 1999; Wang et al., 2020). *Streptococcus*, which is found to harbor hydrogenotrophic microbes, exhibited a significant correlation with the metabolism of hydrogen and carbon dioxide with higher relative abundances in the yak rumen inoculum compared with cattle (Godwin et al., 2014; Wang, 2020).

### 3.2 Higher feed digestibility

The ability to digest plant structural carbohydrates such as cellulose distinguishes ruminants from humankind. Yak rumen microbiota can digest more fibrous feed than cattle with higher feed digestibility (DM, NDF, and ADF digestibility) (Figure 2C, Supplementary Table 3); in particular, there are significant differences in their *in vitro* fermentation comparisons (Figure 2D, Supplementary Table 3). The core members of bacteria cellulose- and hemicellulose-degrading bacteria such as *Fibrobacter succinogenes, Ruminococcus albus*, and *Ruminococcus flavefaciens* are the main cellulose-degrading bacteria, and several bacterial species belonging to the genera *Prevotella, Butyrivibrio*, and *Pseudobutyrivibrio* demonstrate high efficiency in hemicellulose degradation (Perlman et al., 2021). A higher abundance of fiber-degrading bacteria is found in the yak than in cattle rumen under grazing conditions. These include *Ruminococcus, Fibrobacter, Clostridium, Butyrivibrio, Rumenococcus, Treponema*, cellulase-related (GH48, GH5, GH45), and hemicellulase-related (GH44, GH16, GH17, GH11) (Huang, 2013; Mi, 2016; Zhao et al., 2022). Ascomycota or Neocallimastigomycota is the most dominant fungi phylum in the rumen of yaks, regardless of dietary intake (Cao et al., 2010; Yan et al., 2018; Guo et al., 2020). The ability to decompose lignocellulose is enhanced when *Piromyces ruminosae* in yaks secreted polysaccharide hydrolase (xylanase) (Wei et al., 2016). This process may be associated with the fiber-digesting capacity, thus resulting in more structural carbohydrate fermentation and more acetate production when compared with cattle (*Bos taurus*). The carbohydrate-active enzymes (CAZymes) encoded by the microbiome in the rumen play a pivotal role in the digestion of feed in ruminants. Unlike cattle, yaks exhibit a higher relative abundance of CAZymes. Specifically, cellulase, hemicellulase, and PL families are significantly enriched in the rumen microbiome of yaks than in cattle (Zhao et al., 2022), which may help explain the improved fiber degradation in yaks.

### 3.3 Higher short-chain fatty acid concentrations

Higher feed digestibility (DM, NDF, and ADF digestibility) leads to higher energy SCFA concentrations. The rumen microbiome ferments plant materials anaerobically to produce metabolic end products, such as SCFAs while supporting complex food webs (Shabat et al., 2016). Many *in vivo* and *in vitro* studies suggest that yak rumen fermentation can produce more SCFAs (Figures 2E, F, Supplementary Tables 4, 5), especially more acetate and propionate SCFAs, than cattle under *in vitro* rumen fermentations to sustain themselves in harsh environments. *Prevotella* plays an important role in starch and protein degradation and hemicellulose utilization. A high relative abundance of *Prevotella* in the yak is associated with high propionate concentration (Zhang Z. et al., 2016). The increased succinate-producing and utilizing bacterial species such as *Prevotella albensis, Prevotella brevis, Prevotella bryantii, Fibrobacter succinogenes*, and *Succinimonas amylolytica* might also promote propionate concentration in yaks (Zhao et al., 2022). Metagenomic sequencing illustrated the enrichment of yaks' rumen microbial genes in the SCFA production pathways (such as the citrate cycle, TCA cycle, fructose, mannose metabolism, and carbon fixation pathways) (Zhang Z. et al., 2016). Similarly, increased gene and transcript abundances for propionate and butyrate were also observed in high-efficiency ruminants (Kamke et al., 2016). Moreover, yaks demonstrate a higher abundance of glycosyl transferases compared to cattle. The top four microbial KEGG pathways in yaks are pantothenate and CoA biosynthesis, lipopolysaccharide biosynthesis, cysteine and methionine metabolism, and biofilm formation—*Vibrio cholerae* (Zhao et al., 2022).

### 3.4 Higher rumen microbial protein production

Ammonia and amino acids serve as the sources of nitrogen in the rumen, which, in turn, is used by microbes to synthesize microbial proteins. The microbial proteins absorbed from the small intestine account for 40%−80% of the protein needs of the host (Owens and Bergen, 1983). Zhou et al. (2017) used purine derivative excretion estimation and nitrogen isotope techniques to study the differences in nitrogen (N) excretion and retention and urea N recycling in yaks and yellow cattle. The authors found that yaks had low urinary N excretion but higher N retention and urea N recycling to the gut. They also observed that recycled urea N captured by ruminal bacteria was higher in yaks, resulting in higher production of rumen microbial protein synthesis (MCP) than that of yellow cattle (*Bos taurus*) (Zhou et al., 2017, 2018) with the same dietary intake. In addition, *Streptococcus, Akkermansia*, and *uncultured Eubacterium WCHB141_ge* may also regulate the synthesis of MCP during rumen fermentation in yaks (Wang, 2020; Guo et al., 2021). Through the rumen metagenomic sequencing of yaks and cattle, the amino acid pathways (such as valine, leucine, and isoleucine biosynthesis, glycine, serine, and threonine metabolism) and nitrogen metabolism were enriched in the rumen microbiota of yaks compared with those of cattle (Zhang Z. et al., 2016; Zhao et al., 2022), which was likely related to the higher MCP production in yaks (Zhou et al., 2017). Rumen microbial metabolic pathways and metabolites were different than in cattle, and mainly, amino acids were also confirmed later in yaks (Zhao et al., 2022). A total of 11% of amino acids absorbed by the small intestine were derived from protozoa (Shabi et al., 2000), and in the absence of nitrogen in the rumen, protozoa and bacteria synthesized and stored polysaccharides and used them when sufficient nitrogen was available (Dewhurst et al., 2000). Therefore, further research is needed to determine the function of protozoa in yaks during the MCP synthesis.

**Figure 3:**
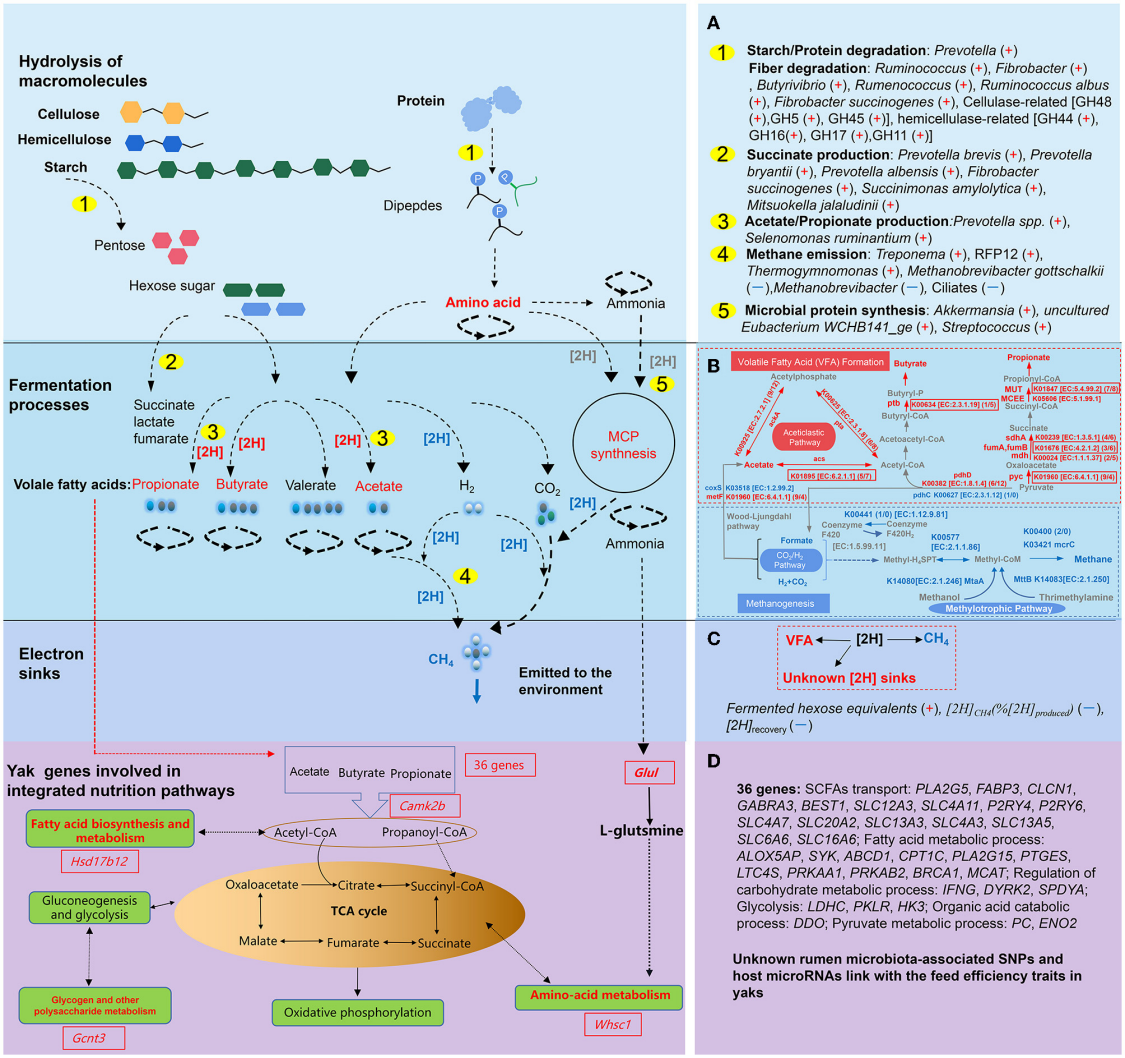
The summarized host gene, rumen microbiota, and the related microbial pathways and hydrogen balance response to the nutritional deprivation in yaks (adapted from Morais and Mizrahi, 2019). **(A)** The microbial community involved in the hydrolysis of macromolecules and fermentation processes. A “+” in the parentheses refers to higher relative abundance, a “–” in the parentheses refers to lower relative abundance in yaks; **(B)** Comparison of methanogenesis and volatile fatty acid (VFA) formation pathways between yaks and cattle (adapted from Zhang Z. et al., 2016). The annotation highlighted in red represents enrichment in the yaks and that in blue represents enrichment in cattle rumen microbiota fermentation. **(C)** Metabolic hydrogen ([2H]) shifting between yak and cattle *in vitro* inocula fermentation. In **(C)** (+) or (–) indicate the enrichment or reduction of the parameters in yaks. **(D)** Host and rumen microbiome interactions on regulating the VFA, microbial protein genesis, and their absorption (adapted from Qiu et al., 2012).

In total, the summarized research on yaks sheds light on the muti-dimensional and intricate rumen ecosystem. However, rumen microorganisms sustain their functionalities and homeostasis through a complex and coordinated process, which involves the establishment of successive food webs with cross-feeding interactions among different rumen microorganisms to provide synergetic services such as SCFAs and MCP for the host (Morais and Mizrahi, 2019). Homeostasis includes interactions between the number and diversity of species, hydrogen shifting, thermodynamics, and corresponding metabolisms of the microorganisms. Among them, microbial interactions were identified as key contributors to the formation of rumen community states, with niche modification emerging as a primary mechanism for their formation. The microbial metabolic cascades, thus, were carried out by the microbial community interactions and provided basic metabolites, which were the outcome of the establishment of numerous parallel trophic chains within each of these structured environments (Morais and Mizrahi, 2019; Mizrahi et al., 2021). To clarify the complex microbial community and its homeostasis, Morais and Mizrahi (2019) proposed categorizing the microbial community into functional groups that could streamline the comparative analysis of rumen communities. This approach is particularly valuable as functional groups have the potential to clarify taxonomic uncertainties resulting from functional redundancies and events of horizontal gene transfer. Mizrahi et al. (2021) additionally highlighted that rumen metabolism could be categorized into three trophic-like levels, representing the broad chemical transformations of plant fiber macromolecules and polymers in broad terms. The degradation and metabolism of cellulose and hemicellulose occurred in the first level. At the second level, in the process of utilizing hexoses and pentoses, specific transporters facilitated the import of soluble sugars into microbial cells. Subsequently, these sugars underwent metabolism through diverse pathways, including the pentose phosphate pathway and the Embden-Meyerhoff-Parnas pathway. In the third level, certain excreted metabolites, including hydrogen, carbon dioxide, lactate, and succinate, underwent additional transformations to yield methane, acetate, propionate, butyrate, and various other byproducts (Mizrahi et al., 2021). Therefore, in yaks, the rumen microbiota composition, the abundance of functional microbiota, and the gene expression were linked with rumen fermentation profiles, which are shown in Figures 3A–C. Although the rumen microbiome has been studied using metatranscriptomic, meta-proteomic, and metabolic methodologies, the study of metaproteome and virome behind the rumen is still in its infancy in yaks.

## 4 Host-rumen microbiome interacts with the feed efficiency-related traits

Evidence indicates a strong connection between host genetics and the microbiome present in the rumen, wherein the genetics of ruminants affects the rumen microbial community structure (Hernandez-Sanabria et al., 2013). The heritability of rumen microbiota has been investigated in ruminants (Li et al., 2019; Wang et al., 2023), and potentially heritable microorganisms could be linked to the phenotype of the host. Some microorganisms own moderate heritability estimates, and they were closely linked with feed efficiency and CH_4_ emissions (Roehe et al., 2016; Difford et al., 2018; Li et al., 2019). For instance, rumen microbiota-associated single-nucleotide polymorphisms (SNPs) played an important role in contributing to the variability observed in feed efficiency traits within the beef cohort (Li et al., 2019), and the relative abundance of certain bacteria and archaea were heritable and exerted their association with CH_4_ production (Difford et al., 2018). The genes annotated within specific genomic regions, such as chromosome 19 at position 3.0–4.0 Mb and chromosome 27 at position 3.0–4.0 Mb, also suggested that the observed associations were directed toward the selective absorption of SCFAs from the rumen, thereby increasing energy availability for the animal (Abbas et al., 2020). However, host genetics may outweigh rumen microorganisms in shaping the related heritable traits, as the variance in CH_4_ production is attributable to both host genetics and the presence of rumen bacteria and archaea. While host genetics account for 21% of the variation, rumen microbes contribute 13% (Difford et al., 2018). Similarly, heritability for sheep body weight could be 39% in genetics, and rumen microbiota explain only 20% of the phenotypic variation (Wang et al., 2023). Although the coevolution of microorganisms with the host might be a mechanism that elucidates varying host genetic effects on distinct rumen microbial taxa, the related heritable rumen microbiota in yaks remains unknown. In addition, host microRNAs, a group of non-coding RNAs and potential molecules, might act as crucial regulators during the metabolic processes and interface the regulation of nutrition, genes, and gut microbes (Malmuthuge et al., 2019; Ojo and Kreuzer-Redmer, 2023), but further research is needed on yaks in this context (Figure 3D).

## 5 Host-rumen microbiome–environment interactions on the dependent animal productivity traits

Microbiome-, host-, and environment-dependent mechanisms contribute to varied performance in the milk and meat production quality of yaks. The microbial metabolite SCFAs, microbial proteins, and the host genes are key molecules that are involved in animal production.

### 5.1 Milk quality

Plants are degraded and fermented by rumen microbes, and the derived microbial metabolite SCFAs and microbial proteins from microbial fermentation are precursors that directly impact the biosynthesis of milk (Flint and Bayer, 2008; Patil et al., 2018). Mostly, milk fatty acids (FAs) are synthesized by the rumen microbiota and exogenous uptake (Parodi, 2004). Acetate and β-hydroxybutyric acid are conveyed to the mammary gland for the synthesis of short and medium-chain FAs, while butyrate is assimilated and transformed into β-hydroxybutyric acid by rumen epithelial cells. The long-chain FAs primarily originate from dietary lipids and adipose tissue (Buitenhuis et al., 2019). The particular plateau environment and rumen microbial services, such as more ruminal SCFAs and more microbial proteins, contribute to the unique nutritional profile of yak milk. Yak milk is known as “natural milk concentrate,” and its protein (4.0–5.9%), fat (5.3–8.8%), lactose (4.0–5.9%), total dry matter content, and ash are significantly higher than in milk of ruminants (Li et al., 2011, 2023).

The microbial metabolites greatly influence the milk quality of yaks. Compared to cow milk, yak milk exhibits considerably elevated levels of essential amino acids, immunoglobulin A (IgA), IgG, and IgM. Immunoglobulin A and IgG concentrations could be approximately 1.5 times higher than those found in human milk (Li et al., 2023). Although the fat content ranges from approximately 5.3% to 8.8% and is the most variable component in yak milk (Ma et al., 2021a), yak milk possesses almost twice the amount of fat compared to Holstein milk (Li et al., 2011). Regarding the fatty acids (FAs) contained in yak milk, it has a reduced concentration of short and medium-chain FAs but an elevated level of long-chain FAs and unsaturated FAs in contrast to Holstein milk (Li et al., 2023). The concentration of conjugated linoleic acid (CLA) in yak milk is significantly greater than that found in cow milk (Zongo et al., 2021). In addition, due to the activity of yak rumen microorganisms and enzymes, numerous polyunsaturated FAs present in fresh forage are hydrogenated and eventually absorbed by the intestine in the form of saturated FAs and trans FAs, leading to their deposition in tissues affecting milk quality (Li et al., 2023). However, little is known about the microbiome-host-dependent mechanisms for yak milk containing high mineral and vitamin content. The main mineral and vitamin content in regular cow milk is lower than in yak milk (Dosek et al., 2007; Ma et al., 2017). The elevated levels of vitamin D in yak milk may be associated with strong ultraviolet radiation at high altitudes. Additionally, the abundant presence of vitamin C and vitamin E imparts strong antioxidant capabilities to yak milk, which may help mitigate oxidative damage induced by the high-altitude harsh environment (Dosek et al., 2007).

### 5.2 Meat quality

The fundamental health of livestock species and traits related to meat quality depend on the symbiotic interactions between the host and microbes (Yeoman and White, 2014). The fatty acid composition of meat can be influenced by rumen microbial fermentation, which supplies precursors for *de novo* fatty acid synthesis (Shingfield et al., 2013). The majority of fatty acids present in ruminant products primarily originate from the metabolism of fat in the rumen rather than from the diet (Toral et al., 2018). Short-chain fatty acids (SCFAs) improve meat quality traits and are conveyed through the host's systemic circulation, reaching extraintestinal organs and exerting broad-range impacts on the host (Tremaroli and Bäckhed, 2012; Koh et al., 2016). The conversion of SCFAs such as acetate, butyrate, and propionate into acetyl-CoA or propynyl-CoA occurs through pathways that include the acetyl-CoA carboxylase (ACSSs) and beta-oxidation. This process results in the generation of ATP, thereby sustaining cellular homeostasis (Dalile et al., 2019). Liu et al. (2021) have highlighted the favorable outcomes of SCFAs derived from the gut microbiota on both muscle and fat tissue, subsequently influencing meat quality. Altering the gut microbiota has the potential to regulate both intramuscular fat deposition and host immunity, contributing to the enhancement of meat quality. Given the overlap among numerous bacterial taxa associated with intramuscular and subcutaneous fat deposits that did not occur, the gut microbiota likely mainly influences adipose accumulation through separate adipogenic pathways (Krause et al., 2020). Therefore, understanding the interaction between the host and rumen microbiota is essential for developing knowledge-based strategies that improve both animal meat quality and host health. Concerning the quality of yak meat, it features a reduced fat yet a higher protein percentage and is abundant in essential amino acids, fatty acids, and minerals when compared to commercial beef meat from low-altitude regions (Yin et al., 2009; Wan et al., 2012).

### 5.3 Host genes are involved in milk and meat production

Yak milk is characterized by its elevated levels of fat and protein, and the gene expression patterns are also related to its synthesis. For instance, the genes associated with the uptake of fatty acids from the blood (*CD36* and *LPL*), intracellular fatty acid activation of long and short-chain fatty acids (*ACSL1, ACSS1*, and *ACSS2*), intracellular fatty acid transport (*FABP3*), triacylglycerol synthesis (*LPIN1, AGPAT6*, and *GPAM*), lipid droplet formation (*BTN1A1, PLIN2*, and *XDH*), desaturation (*SCD*), and ketone body utilization (*BDH1* and *OXCT1*) exhibit significant upregulation during yaks' lactation. In particular, compared to the upregulation levels in dairy cows, the processes of triacylglycerol synthesis (*GPAM, AGPAT6*, and *LPIN1*) and intracellular *de novo* fatty acid synthesis (*ACACA, ACSS2*, and *FABP3*), which potentially orchestrate as components within the gene network controlled by *SERBF1* during milk fat synthesis, exhibit a higher degree of activation (Lee et al., 2017). Moreover, in the lactation cycle of yaks, the highest expression of certain milk fat genes (such as *XDH* and *FABP3*) in mammary tissue occurs earlier than observed in dairy cows (Yuan et al., 2020). *FASN* is one of the genes with high expression levels in the yak mammary gland and subcutaneous fat and has the potential to be a genetic marker in breeding programs to enhance the milk fat content and total milk solid levels (Shi et al., 2019). As to the meat quality, under the nutritional deprivation environment, comparative transcriptomics of yak and cattle show that the genes differentially expressed in tissues, including skeletal muscles, are significantly enriched in the energy metabolism-related process (Tang et al., 2017; Ma et al., 2021b). Another comparative gene expression study performed based on subcutaneous adipose tissues showed that introducing yak genes into cattle breeds by hybridization dramatically changed the expression patterns of genes related to fatty acid biosynthesis and catabolism and improved the yield and quality of meat (Song et al., 2019), highlighting the unique genetic basis of nutrition accumulation in yaks. Similarly, unique patterns of adaptations related to meat production have also been revealed by population genetics in yak. Furthermore, the genomic copy number variations of the *CHKB* and *CHRM3* genes, which are detected in domesticated yak populations using whole genome resequencing data (Zhang X. et al., 2016), are significantly associated with improved growth traits such as higher body weight and greater chest girth (Goshu et al., 2019, 2020). Intriguingly, these copy number variations have not been detected in cattle breeds (Yue et al., 2014; Zhang et al., 2014), implying the distinct genetic basis of energy storage, growth, and development in yaks.

In summary, these findings provide insights into the rumen microbiome-dependent traits that interact with the metabolism, environment, and animal production, demonstrating the host-rumen microbiome-environment as a whole in response to environmental stress. However, the interactions of host-rumen microbiome-environment on the yak productivity traits need to be clarified.

## 6 Implications

### 6.1 Integrating host-gut microbe-environment interactions into understanding animal performance and improving the systematic management

**Figure 4:**
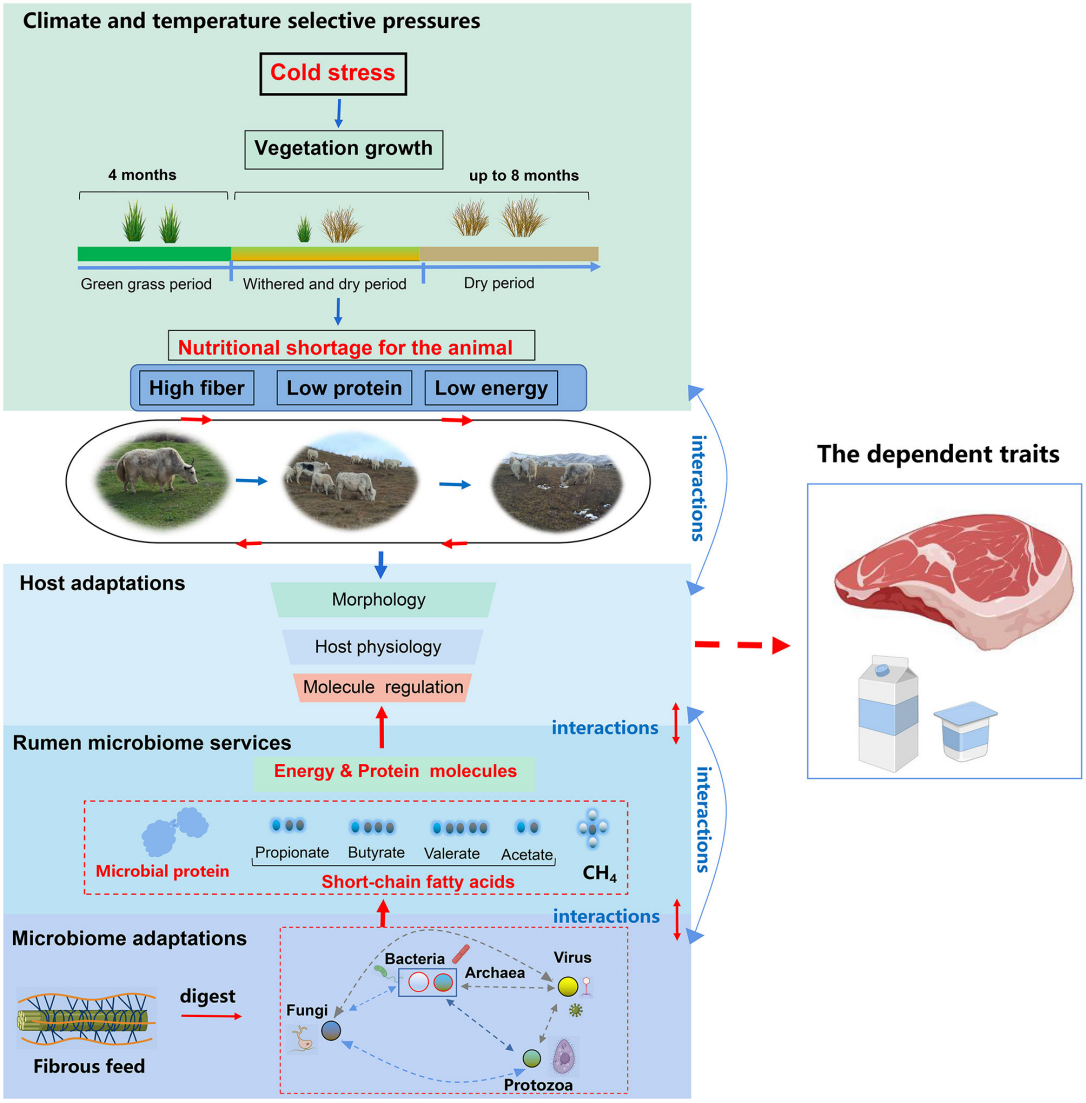
Hierarchical structure of the environment and host-gut microbe interactions.

The proposal by Kohl (2018) to include host-microbe interactions in the established field of animal comparative physiology opened exciting research opportunities for both fields. However, our knowledge about the mechanistic bases of host-environment linkages and how they ultimately benefit the organism in adapting to changes to serve the ecosystem is limited (Hutchinson, 1957; Angilletta and Sears, 2011). The adaptation of yak to environmental stress sheds light on understanding some of these linkages. The yak's adaptation to the stress of nutritional shortage is directly related to environmental services (Figure 4). By coping with the harsh environment, the animals themselves show multi-faceted synergism. At the host level, yaks possess genes that are co-regulated, embodied in the regulation of traits ranging from molecules, cells, tissues, organs, and systems (Dalziel et al., 2009; Shao et al., 2010; Qiu et al., 2012; Jing et al., 2022). In addition to tissue morphology, the host's metabolism processes (energy metabolism, nitrogen metabolism, etc.) and gene regulation interact with each other (Qiu et al., 2012). Mediated by the host, gut microbes process their niches and reciprocally provide services to sustain a symbiotic relationship with the host (Figure 4). Adaptation, thus, is a consequence of the interactions at various hierarchical levels including climate pressure, nutritional pressure, host, and rumen microbiome and its services to the yaks. These selective pressures of the host combine with the environment to affect the gut microbe, which ultimately effectuates the adaptation of the microorganism-host symbionts to the environment.

From the ecological systematic balance, understanding the host-rumen-environment linkages is beneficial for further animal management. The host-environment linkages may provide a means for devising improved animal management strategies. For instance, under the stress of cold, with low temperature and insufficient forage on the Qinghai-Tibetan Plateau, the yak loses body weight (Long et al., 1999b) and increases forage intake from the grassland to avoid the nutritional deprivation, increasing the pressures on the grassland. Better management, therefore, would involve reducing the selective pressure of grassland to increase its biomass productivity or directly decreasing the number of grazing yaks. Artificial seeding or supplementary feeding of fodder for the animals are approaches to relieve the pressure. However, the animal management strategy should also focus on the forage (biomass, fatty acid content, richness and diversity, etc.) from the pasture. Since the forage from the pasture provides a certain level of nutrients, they can influence the quality of animal products. For instance, the concentrations of total conjugated linoleic acid (CLA), CLA isomer c9t11, and CLA isomer t10c12 in the milk of grazing yaks were significantly higher during the peak grass stage compared to the dry grass stages (Pan et al., 2021). Ruminants grazing artificial pasture led to an elevation in the levels of polyunsaturated fatty acids (PUFAs) in meat, particularly an increase in n3 PUFA concentrations and a decrease in the n6/n3 ratio (Wang et al., 2021). Ruminants fed with alfalfa (*Medicago sativa L*.) exhibited significantly higher contents of saturated fatty acids such as C14:0, C16:0, and C18:0 in meat (Wang et al., 2022). Therefore, maintaining the quality of the grass is also crucial for healthy animal products.

The gut-environment linkages are also vital in understanding adaptation (Boyce et al., 2020) and devising improved animal management strategies. Precise supplemental feeding methods for the yaks during warm and cold seasons are advisable since the rumen microbiota can utilize low levels of dietary nitrogen better than the high (Zhou et al., 2017; Hao, 2019), and excessively high concentrations of protein, which may result in unnecessary waste and environmental pollution. Moreover, the correlation between forage and milk fatty acids is significantly affected by the biohydrogenation occurring in the yak rumen alongside the particular community of rumen microorganisms (Li et al., 2023). The intestinal microbiota of yaks determine their feed quality, and studies have shown that yaks can selectively intake certain plants. Its consumption varies with different pastures and seasons (Ding, 2007; Guo et al., 2021), which is essential for regulating the flavor and quality of milk and animal products. From the gut-environment linkages, microbiota also provides regulation services that help to maintain a stable condition for the Qinghai-Tibetan Plateau and may prevent the plants from aggressive growth. Many plants produce compounds to deter herbivores and include chemical compounds such as alkaloids, glycosides, terpene, benzene, and some secondary metabolites such as essential oils, tannins, and nitrate compounds (Long et al., 1999a; Hart et al., 2008). Gut microbiota could detoxicate “biohazardous waste” that is poisonous or other undesirable ingesta. The long-term association between the gut microbiome of yaks and undesirable plants is perhaps indicative of the development of strategies adapted by yaks to benefit from these plants. It has been reported that yaks can digest toxic plants during harsh winters (Guo et al., 2021). The ingested plant tannins bind themselves to microbial proteins and prevent them from being degraded in the rumen, thereby forming more proteins for intestinal absorption. This may have helped yaks not suffer from nitrogen deficiency (Long et al., 2003). Studying such an adaptive behavior can suggest new prospects for improving animal production (for instance, incorporating host-gut-environment linkages into breeding strategies as a whole), understanding the role of rumen microbiota systematically that shape the feed efficiency and withstanding the body homeostasis or co-evolve with the host, and developing more tools to decipher and manipulate the microbes.

The latest “omics” techniques propose to integrate a database to optimize the microbiome fermentation traits, including flavonoids (Oh et al., 2017; Morales et al., 2018), essential oils (Cobellis et al., 2016), nitro-compounds (Latham et al., 2016), or other secondary metabolites fermentation. Although the rumen microbial community composition and fermentation profiles are attributable predominantly to diet, with the host having a lesser influence (Henderson et al., 2015), integrating host-gut microbe interactions into understanding the host-environment linkages might favor the establishment of a predictive theory of the niche in organismal biology. In this context, the yak is an excellent animal model to study the influences of environmental factors on the host and the relationship between the host and rumen microbiome.

### 6.2 The potential to understand the host-gut microbe-environment linkages

Understanding these linkages can augment our capacity to anticipate and predict relationships among hosts, gut microbes, and environments over space and time (Angilletta and Sears, 2011; Kohl, 2018). A modeling approach, thus, could advance prediction with a combination of experiments for validation by integrating and synthesizing biological principles from the bottom up. On the other hand, an experimental approach involving reduction and analysis is from the top down. Generating such predictions necessitates a comprehensive collection of models that elucidate how hosts interact with their environments and the reasons behind their specific interactions. The poor coordination between theoretical and empirical activities and previous models fails to explain variations in fundamental ecological niches within and among organisms (Angilletta and Sears, 2011). Focusing on the ecological foundations of the rumen microbiota could lead to an enhanced understanding of both functions and unexplored fermentation pathways of the rumen in yaks (Huws et al., 2018; Solomon et al., 2022), improving the understanding and predicted linkages between host and environments and the integration of mathematical models and crucial experiments in a manner that has worked for biological disciplines (Gilarranz et al., 2017). With the development of research tools and methodology, mathematical models (Dalziel et al., 2009) such as network-based approaches (Dee et al., 2017; Huws et al., 2018) and deep learning algorithms. These are better choices for systematic processing and quantification, helping us to better understand complex systems and their related connections. Although comprehensive network-based approaches have clarified the connections in microbial communities (Adai et al., 2004), large-scale, integrative models have yet to be developed (Huws et al., 2018). A systematic analysis of animals (*in vivo* or between different host systems) and their living environment is essential to better manage the performance of animals and their habitats. This involves integrating various layers of interactive networks, including living organisms, vegetation, landscapes, soils, gut microbes, and other layers.

## 7 Conclusion

The host gene regulation, host gene metabolism, and the rumen microbial services of yaks to survive in an extreme environment provide a basic understanding of the animal adaptability mechanisms and performance. In this review, a hierarchical model of the adaptability between the host, the environment, and the related host-rumen microbiome-environment interactions was integrated and proposed. It offers more solutions for the regulation of rumen microorganisms and is mutually beneficial for the hosts as well as the microorganisms. To better understand the relationship between organisms and the environment, it is proposed that multi-level interactions and primary determinants be highlighted and clarified in systematic biology research. It would be beneficial for sustainable animal production management and systematized regulation, but further research is required.

## Author contributions

WW: Writing – original draft, Conceptualization, Funding acquisition, Investigation, Methodology, Resources, Software. YD: Investigation, Writing – original draft. WG: Conceptualization, Writing – original draft. XZ: Investigation, Writing – original draft. AD: Writing – review & editing. SB: Investigation, Writing – original draft. LD: Investigation, Writing – review & editing. XC: Funding acquisition, Writing – review & editing, Conceptualization. RL: Conceptualization, Funding acquisition, Supervision, Writing – review & editing.
