# Supplementary material for: Linkages between rumen microbiome, host, and environment in yaks, and their implications for understanding animal production and management
Source: Front Microbiol. 2024 Jan 29;15:1301258. doi: 10.3389/fmicb.2024.1301258 (PMC10860762; doi:10.3389/fmicb.2024.1301258)
Supplement: Supplementary file 1 [file Data_Sheet_1.doc]

1. **Calculation of the** **rumen fermentation profiles from the comparisons between yak and cattle of each measure**

The rumen fermentation profiles of individual measures for the corresponding situations were assessed by comparing the results of yak and cattle groups using the following formula:

$\text{E}_{\text{m}}=(\frac{\text{ER}_{\text{yak}}}{\text{ER}_{\text{cattle}}}-1)\times100\%$ (1)

where *E_m_* is response ratio, *ER_yak_* is the yak experimental group with measures, and *ER_cattle_* is the cattle group. Thus, a negative or positive *E*_m_ value indicates that the selected measure can reduce or increase the effect, respectively. The median *E*_m_ values for each measure were calculated using an analytical approach adapted from Benayas et al. (2009) and Tuomisto et al. (2012) The normality of the data was tested using the Kolmogorov-Smirnov test. Not all of the *E*_m_s for each effect measure were normally distributed; therefore, the Wilcoxon Signed-Rank test was used to determine if the median *E*_m_s were significantly different from zero when there were sufficient results for specific measures (Wang et al., 2018). SPSS 22.0 software was used for the statistical analyses.

**Supplementary Table 1** The methane emission comparisons between yak and cattle under differing methods collected from differing experiments

| Animal | Age^1^ | Body weight (kg) | Methods | Observations | Feed conditions | DMI Intake (kg/d) | CH_4_ emission (g/d) | References^2^ |
| --- | --- | --- | --- | --- | --- | --- | --- | --- |
| Yak | 3 y | 175 ± 10.7 | SF_6_ | 12 | Alpine meadow grass | 3.78 | 81.40 | Ding et al. (2010) |
| Yak | 2 y 3 m | 94.56 ± 3.9 | SF_6_ | 6 | Grazing | —— | 71.45 | Hao (2019) |
|  | 2 y 3 m | 95.01±4.1 | SF_6_ | 6 | Supplementary feeding | —— | 74.17 |  |
|  | 2 y 3 m | 94.56 ± 3.9 | SF_6_ | 6 | Grazing | —— | 2.05 g/kg/d BW^0.75^ |  |
|  | 2 y 3 m | 95.01 ± 4.1 | SF_6_ | 6 | Supplementary feeding | —— | 1.98 g/kg/d BW^0.75^ |  |
| Female yak | 1 - 2 y | —— | Mask | 4 | Alpine meadow grass | —— | 54.59 | Xue et al. (2017) |
| Female yak | 3 - 4 y | —— |  | 4 |  | —— | 80.48 |  |
| Female yak | 5 - 6 y | —— |  | 4 |  | —— | 84.45 |  |
| Male Angus | —— | 271 ± 22 | Point-source | 18 | Rhodes grass, Leucaena | —— | 141.00 | McGinn et al. (2011) |
| Beef cattle | —— | 222 ± 3.8 | Chamber | 9 | Pasture forage | 4.0 ± 0.19 | 114.30 | Tomkins et al. (2011) |
| Dairy heifers | 8 m | 230 ± 6 | SF_6_ | 12 | Alfalfa and ryegrass pasture | 9.15 ± 2.67 | 186.00 | Hammond et al. (2015) |
| Yak |  |  | Model estimation |  |  |  | 99.70 | Feng et al. (2012) |
| Dairy heifer |  |  |  |  |  |  | 111.60 |  |
| Beef cattle |  |  |  |  |  |  | 122.00 |  |
| Draught cattle |  |  |  |  |  |  | 136.50 |  |
| Continued | | | | | | | | |
| Yak | 3 y | 156 ± 8 | Chamber | 6 | Natural grass (voluntary intake) | 8.03 | 22.56 | Shi (2022) |
| Cattle | 3 y | 158 ± 7 |  | 6 |  | 9.51 | 26.58 |  |
| Yak | 3 y | 156 ± 8 |  | 6 | Oat grass (voluntary intake) | 8.05 | 23.07 |  |
| Cattle | 3 y | 158 ± 7 |  | 6 |  | 10.28 | 30.22 |  |

^1^ y: year; m: month.

^2^ Feng et al. (2012) is the same experiment and with the same estimation; Shi (2022) is the same experiment and with the same diet. Their data were used to compare the rumen fermentation profiles between yak and cattle groups using formula 1.

**Supplementary Table 2** The *in vitro* methane production comparisons between yak and cattle collected from differing experiments

| Animal | Age^1^ | Body weight (kg) | Methods | Observations | Feed conditions/ Substrate | Dose (g) | CH_4_ emission (mL) | References^2^ |
| --- | --- | --- | --- | --- | --- | --- | --- | --- |
| Yak | 4 y | 201±11 | *In vitro* gas technique | 3 | Grazing/ Oat hay | 0.4 | 4.53 | Zhang et al. (2016) |
| Cattle | 4 y | 230±17 |  | 3 |  | 0.4 | 8.46 |  |
| Yak | —— | 192±12 | *In vitro* gas technique | 3 | Housing/ Mixed diet | 20 | 4.70 mmol/d | Mi et al. (2017) |
| Cattle | —— | 192±12 |  | 3 |  | 20 | 6.20 mmol/d |  |
| Yak | —— | 215 ± 13 | *In vitro* gas technique | 6 | Housing/ Oat hay | 0.4 | 12.35 | Wang (2020) |
| Cattle | —— | 231 ±12 |  | 6 |  | 0.4 | 15.66 |  |
| Yak | 4 y | 201±11 | *In vitro* gas technique | 8 | Green grass period/ Oat hay | 0.4 | 13.17 |  |
| Cattle | 4 y | 230±17 |  | 8 |  | 0.4 | 12.98 |  |
| Yak | 4 y | 201±11 | *In vitro* gas technique | 8 | Withered and yellow period/ Oat hay | 0.4 | 15.22 |  |
| Cattle | 4 y | 230±17 |  | 8 |  | 0.4 | 18.78 |  |
| Yak | 4 y | 201±11 | *In vitro* gas technique | 8 | Withered period/ Oat hay | 0.4 | 11.76 |  |
| Cattle | 4 y | 230±17 |  | 8 |  | 0.4 | 14.65 |  |

^1^ y: year; m: month.

^2^ These data were all from the same experiment and with the same diet and were used to compare the rumen fermentation profiles between yak and cattle groups using formula 1. In the research of Zhang et al. (2016) and Wang (2020), the *in vitro* methane productions were determined at 48 h or 72 h, respectively.

**Supplementary Table 3** The feed digestibility comparisons between yak and cattle under differing determined methods collected from differing experiments

| Animal | Age^1^ | Body weight (kg) | Method | Observations | Feed conditions /Substrate | Intake (g DM/ kg BW^0.75^)/ Dose (g) | Feed DM digestibility (%)^2^ | NDF digestibility (%)^2^ | ADF digestibility (%)^2^ | References^3^ |
| --- | --- | --- | --- | --- | --- | --- | --- | --- | --- | --- |
| Yak | —— | 169 ± 6.0 | Voluntary intake | 3 | ——/ Oat hay | 36.9 | 64.8 | 63.1 | 54.6 | Wang (2009) |
| Cattle | —— | 121 ± 8.0 |  | 3 | ——/ Oat hay |  | 62.4 | 57.3 | 57.1 |  |
| Yak | —— | 169 ± 6.0 | Voluntary intake | 3 | ——/ Oat hay | 55.4 | 62.6 | 55.7 | 56.9 |  |
| Cattle | —— | 121 ± 8.0 |  | 3 | ——/ Oat hay |  | 61.4 | 46.4 | 55.6 |  |
| Yak | 3 y | 156 ± 8 | Voluntary intake | 6 | Natural grass feed | 2.81 kg/d | 56.3 | 63.8 | 62.0 | Shi (2022) |
| Cattle | 3 y | 158 ± 7 | Voluntary intake | 6 | Natural grass feed | 2.79 kg/d | 51.5 | 56.8 | 53.3 |  |
| Yak | 3 y | 200 ± 15 | *In vitro* gas technique | 4 | Grazing/ Astragalus mahoschanicus | 0.40 | 69.9 | 25.42 | 19.74 | Bi (2019) |
| Cattle | 3 y | 200 ± 15 |  | 4 |  | 0.40 | 70.86 | 22.58 | 18.46 |  |
| Yak | 3 y | 200 ± 15 | *In vitro* gas technique | 4 | Grazing/ Astragalus *polycladus* | 0.40 | 67.8 | 44.66 | 40.87 |  |
| Cattle | 3 y | 200 ± 15 |  | 4 |  | 0.40 | 64.61 | 37.12 | 33.49 |  |
| Yak | 3 y | 200 ± 15 | *In vitro* gas technique | 4 | Grazing/ Astragalus przewalskii | 0.40 | 63.98 | 28.33 | 21.12 |  |
| Cattle | 3 y | 200 ± 15 |  | 4 |  | 0.40 | 62.55 | 25.25 | 16.07 |  |
| Yak | 3 y | 200 ± 15 | *In vitro* gas technique | 4 | Grazing/ Astragalus floridus | 0.40 | 66.63 | 42.32 | 36.84 |  |
| Cattle | 3 y | 200 ± 15 |  | 4 |  | 0.40 | 61.08 | 30.35 | 26.18 |  |
| Yak | 3 y | 200 ± 15 | *In vitro* gas technique | 4 | Grazing/ Oat hay | 0.40 | 58.98 | 34.58 | 26.84 |  |
| Cattle | 3 y | 200 ± 15 |  | 4 |  | 0.40 | 58.00 | 26.96 | 20.76 |  |
| Yak | 4 y | 215 ± 13 | *In vitro* gas technique | 6 | Housing/ Oat hay | 0.40 | 61.92 | 41.8 | 41 | Wang (2020) |
| Cattle | 4 y | 231 ±12 |  | 6 |  | 0.40 | 56.58 | 37.5 | 35.5 |  |
| Yak | 4 y | 201 ± 11 | *In vitro* gas technique | 8 | Green grass period/ Oat hay | 0.40 | 56.79 | 35.1 | 33.6 |  |
| Cattle | 4 y | 230 ± 17 |  | 8 |  | 0.40 | 49.91 | 30.2 | 28.5 |  |
| Continued | | | | | | | | | |  |
| Yak | 4 y | 201 ± 11 | *In vitro* gas technique | 8 | Withered and yellow period/ Oat hay | 0.40 | 61.30 | 40.1 | 35.4 |  |
| Cattle | 4 y | 230 ± 17 |  | 8 |  | 0.40 | 57.08 | 38.0 | 32.1 |  |
| Yak | 4 y | 201 ± 11 | *In vitro* gas technique | 8 | Withered period/ Oat hay | 0.40 | 56.27 | 34.1 | 31.2 |  |
| Cattle | 4 y | 230 ± 17 |  | 8 |  | 0.40 | 52.67 | 27.3 | 27.3 |  |

^1^ y: year; m: month.

^2^ DM: dry matter; NDF: neutral detergent fiber; ADF: Acid detergent fiber.

^3^ These data were all from the same experiment and with the same diet and were used to compare the rumen fermentation profiles between yak and cattle groups using formula 1.

**Supplementary Table 4** The rumen fermentation VFA profile (*in vivo*) comparisons between yak and cattle collected from differing experiments

| Animal | Age^1^ | Feed conditions | Observations | Parameters^2^ | | | | | References^3^ |
| --- | --- | --- | --- | --- | --- | --- | --- | --- | --- |
|  |  |  |  | Acetate | Propionate | Butyrate | TVFA | AP ratio |  |
| Yak | 4 ± 0.6 y | Summer grazing | 4 | 58.56 | 12.13 | 9.03 | 81.69 | 4.82 | Huang et al. (2012) |
| Cattle | 4 ± 0.4 y |  | 4 | 42.57 | 7.35 | 7.25 | 58.41 | 5.79 |  |
| Yak | 3 y | Summer grazing | 3 | 55.44 | —— | —— | 77.92 | —— | Wang et al. (2012) |
| Cattle | 3 y |  | 3 | 35.41 | —— | —— | 54.58 | —— |  |
| Yak | 3.5 - 4 y | Summer grazing | 3 | 59.89 | 12.06 | 9.28 | 83.24 | 4.96 | Huang et al. (2016) |
| Cattle | 3.5 - 4 y |  | 3 | 42.77 | 7.15 | 7.02 | 58.29 | 5.98 |  |
| Yak | 4 y | 2 h after grazing in summer | 3 | 63.20 | 14.30 | 10.50 | 89.60 | 4.42 | Shi et al. (2019) |
| Cattle | 4 y |  | 3 | 52.50 | 12.50 | 10.60 | 77.00 | 4.20 |  |
| Yak | 4 y | 2 h after grazing in winter | 3 | 34.30 | 6.10 | 4.00 | 42.80 | 5.62 |  |
| Cattle | 4 y |  | 3 | 29.00 | 7.00 | 3.80 | 41.00 | 4.14 |  |
| Yak | 2.5 y | 10.32 Dietary N (g/kg DM) | 4 | 43.27 | 14.84 | 5.08 | 64.97 | 2.92 | Zhou et al. (2018) |
| Cattle | 2.5 y |  | 4 | 38.50 | 13.05 | 5.36 | 58.74 | 2.95 |  |
| Yak | 2.5 y | 19.49 Dietary N (g/kg DM) | 4 | 43.57 | 15.38 | 6.01 | 66.94 | 2.83 |  |
| Cattle | 2.5 y |  | 4 | 41.07 | 14.18 | 4.73 | 60.83 | 2.90 |  |
| Yak | 2.5 y | 28.5 Dietary N  (g/kg DM) | 4 | 42.31 | 14.88 | 4.89 | 64.56 | 2.84 |  |
| Cattle | 2.5 y |  | 4 | 41.01 | 14.58 | 3.62 | 61.71 | 2.81 |  |
| Yak | 2.5 y | 37.58 Dietary N (g/kg DM) | 4 | 44.40 | 14.11 | 4.62 | 65.98 | 3.15 |  |
| Cattle | 2.5 y |  | 4 | 40.37 | 13.16 | 4.32 | 60.70 | 3.07 |  |
| Yak | 4 - 6 y | grazing | 10 | 52.63 | 10.08 | 6.00 | 70.08 | 5.22 | Zhao (2019) |
| Cattle | 4 - 6 y |  | 10 | 57.66 | 9.79 | 5.26 | 74.00 | 5.89 |  |
| Continued | | | | | | | | | |
| Yak | 4 y | Spring | 10 | 50.84 | 9.45 | 4.30 | 65.71 | 5.38 | Li (2020) |
| Cattle | 4 y |  | 10 | 43.75 | 8.73 | 4.56 | 58.35 | 5.01 |  |
| Yak | 4 y | Summer | 10 | 55.22 | 11.18 | 5.34 | 80.44 | 4.94 |  |
| Cattle | 4 y |  | 10 | 52.27 | 10.71 | 4.43 | 75.77 | 4.88 |  |
| Yak | same age | cold season | 10 | 42.48 | 9.59 | 4.78 | 55.90 | 4.43 | Li et al. (2021) |
| Cattle |  |  | 10 | 35.48 | 10.58 | 5.52 | 47.73 | 3.35 |  |
| Yak | 3 y | Spring grazing | 6 | 48.66 | 9.18 | 7.89 | 69.03 | 5.30 | Guo (2021) |
| Cattle | 3 y |  | 6 | 48.11 | 10.90 | 6.14 | 67.43 | 4.41 |  |
| Yak | 3 y | Summer grazing | 6 | 48.22 | 10.66 | 6.69 | 68.31 | 4.52 |  |
| Cattle | 3 y |  | 6 | 63.75 | 13.35 | 6.46 | 86.36 | 4.78 |  |
| Yak | 3 y | Autumn grazing | 6 | 54.57 | 11.91 | 3.97 | 73.07 | 4.58 |  |
| Cattle | 3 y |  | 6 | 48.41 | 10.59 | 5.20 | 62.96 | 4.57 |  |
| Yak | 3 y | Winter grazing | 6 | 42.91 | 9.29 | 4.05 | 57.52 | 4.62 |  |
| Cattle | 3 y |  | 6 | 52.72 | 10.81 | 5.05 | 68.57 | 4.88 |  |

^1^ y: year; m: month.

^2^ TVFA: total volatile fatty acid. AP ratio = acetate to propionate molar ratio.

^3^ These data were all from the same experiment and with the same diet and were used to compare the rumen fermentation profiles between yak and cattle groups using formula 1.

**Supplementary Table 5 The rumen fermentation VFA profile (*in vitro*) comparisons between yak and cattle collected from differing experiments**

| Animal | Age^1^ | Feed conditions | Methods | Run | Replicates of each run | Dose (g) | Substrate | Parameters^2^ | | | |  | References^3^ |
| --- | --- | --- | --- | --- | --- | --- | --- | --- | --- | --- | --- | --- | --- |
|  |  |  |  |  |  |  |  | Acetate | Propionate | Butyrate | TVFA | AP ratio |  |
| Yak | ~3 y | Grazing | *In vitro* gas technique | 2 | 3 | 0.4 | Oat hay | 62.74 | 21.05 | 9.95 | 97.83 | 2.98 | Zhang et al. (2016) |
| Cattle | ~3 y |  |  | 2 | 3 | 0.4 |  | 58.24 | 19.46 | 7.11 | 88.65 | 2.99 |  |
| Yak | —— | Housing | *In vitro* gas technique | —— | 3 | 20 | Low N feed | 23.02 | 14.81 | 6.38 | 46.60 | 1.55 | Mi et al. (2017) |
| Cattle | —— |  |  | —— | 3 | 20 |  | 19.08 | 12.26 | 4.61 | 38.10 | 1.56 |  |
| Yak | ~4 y | Housing | *In vitro* gas technique | 2 | 3 | 0.4 | Oat hay | 48.20 | 19.10 | 5.93 | 76.00 | 2.52 | Wang (2020) |
| Cattle |  |  |  | 2 | 3 | 0.4 |  | 40.30 | 15.10 | 5.27 | 62.20 | 2.67 |  |
| Yak | ~4 y | Green grass period | *In vitro* gas technique | 2 | 4 | 0.4 | Oat hay | 53.64 | 18.40 | 10.80 | 88.60 | 2.92 |  |
| Cattle |  |  |  | 2 | 4 | 0.4 |  | 42.13 | 15.89 | 7.28 | 68.72 | 2.65 |  |
| Yak | ~4 y | Withered and yellow period | *In vitro* gas technique | 2 | 4 | 0.4 | Oat hay | 51.70 | 18.01 | 9.36 | 83.59 | 2.87 |  |
| Cattle |  |  |  | 2 | 4 | 0.4 |  | 46.58 | 15.51 | 8.08 | 73.55 | 3.00 |  |
| Yak | ~4 y | Withered period | *In vitro* gas technique | 2 | 4 | 0.4 | Oat hay | 47.82 | 15.06 | 6.03 | 71.19 | 3.18 |  |
| Cattle |  |  |  | 2 | 4 | 0.4 |  | 39.78 | 14.13 | 8.05 | 64.26 | 2.81 |  |

^1^ y: year; m: month.

^2^ TVFA: total volatile fatty acid. AP ratio = acetate to propionate molar ratio.

^3^ These data were all from the same experiment and with the same diet and were used to compare the rumen fermentation profiles between yak and cattle groups using formula 1.

**References**

Benayas, J. M. R., Newton, A. C., Diaz, A., and Bullock, J. M. (2009). Enhancement of biodiversity and ecosystem services by ecological restoration: a meta-analysis. *Science*, 325, 1121-1124. doi: 10.1126/science.1172460

Bi, S. S. (2019). *Effects of Astragalus plants on in vitro fermentation characteristics of yak and cattle* (In Chinese)*.* M.S. Dissertation. Lanzhou: Lanzhou University.

Ding, X. Z., Long, R. J., Kreuzer, M., Mi, J. D., and Yang, B. (2010). Methane emissions from yak (*Bos grunniens*) steers grazing or kept indoors and fed diets with varying forage: concentrate ratio during the cold season on the Qinghai-Tibetan Plateau. *Anim. Feed Sci. Technol.* 162, 91-98. doi: 10.1016/j.anifeedsci.2010.09.007

Feng, Y. L., Li, S. L., Zhao, G. Y., Zhang, X. M., Mo, F., and Han, J. F. (2012). Estimation of Methane Emissions by Cattle in China. *CHIN. J. Anim. Nutr.* 24: 1 - 7.

Guo, W. (2021). *The development model of microbiota in the rumen of grazing yak* (In Chinese). Ph.D Dissertation. Lanzhou: Lanzhou University.

Hammond, K. J., Humphries, D. J., Crompton, L. A., Green, C., and Reynolds, C. K. (2015). Methane emissions from cattle: estimates from short-term measurements using a GreenFeed system compared with measurements obtained using respiration chambers or sulphur hexafluoride tracer. *Anim. Feed Sci. Technol.* 203, 41-52. doi: 10.1016/j.anifeedsci.2015.02.008

Hao, L. Z. (2019). *Effect of supplementary feeding in warm season on meat quality of yaks and underlying mechanisms* (In Chinese). Ph.D Dissertation. Lanzhou: Lanzhou University.

Huang, X. D., Martinez-Fernandez, G., Padmanabha, J., Long, R., Denman, S. E., and McSweeney, C. S. (2016). Methanogen diversity in indigenous and introduced ruminant species on the Tibetan Plateau. *Archaea*. 2016. doi: 10.1155/2016/5916067

Huang, X. D., Tan, H. Y., Long, R., Liang, J. B., and Wright, A. D. G. (2012). Comparison of methanogen diversity of yak (*Bos grunniens*) and cattle (*Bos taurus*) from the Qinghai-Tibetan plateau, China. *BMC Microbiol*. 12, 237. doi: 10.1186/1471-2180-12-237

Li, C. (2020). *Seasonal changes in grazing behavior and rumen microbiota of yak and cattle* (In Chinese). M.S. Dissertation. Lanzhou: Institute of Husbandry and Pharmaceutical Sciences.

Li, C., Aunm, A. A., Zhang, J. B., Liang, Z. Y., Ding, X. Z., and Yan, P. (2021). Comparative study of grazing behavior, serum biochemical indexes, and rumen fermentation parameters of yaks and cattle in the cold season (In Chinese). *Acta Pratac. Sin.* 30, 162. doi: 10.11686/cyxb2020225

McGinn, S. M., Turner, D., Tomkins, N., Charmley, E., Bishop-Hurley, G., and Chen, D. (2011). Methane emissions from grazing cattle using point-source dispersion. *J. Environ. Qual*. 40, 22-27. doi: 10.2134/jeq2010.0239

Mi, J., Zhou, J., Huang, X., and Long, R. (2017). Lower methane emissions from yak compared with cattle in rusitec fermenters. *PLoS One* 12, e0170044. doi: 10.1371/journal.pone.0170044

Shi, F., Wang, H., Degen, A. A., Zhou, J., Guo, N., Mudassar, S., et al. (2019). Rumen parameters of yaks (*Bos grunniens*) and indigenous cattle (*Bos taurus*) grazing on the Qinghai‐Tibetan Plateau. *J. Anim. Physiol. Anim. Nutr. (Berl)* 103, 969-976. doi: 10.1111/jpn.13095

Shi, F. (2022). *Rumen microbiological mechanisms of efficient energy and nitrogen utilization in yak* (In Chinese). Ph.D Dissertation. Lanzhou: Lanzhou University.

Tomkins, N. W., McGinn, S. M., Turner, D. A., and Charmley, E. (2011). Comparison of open-circuit respiration chambers with a micrometeorological method for determining methane emissions from beef cattle grazing a tropical pasture. *Anim. Feed Sci. Technol.* 166, 240-247. doi: 10.1016/j.anifeedsci.2011.04.014

Tuomisto, H. L., Hodge, I. D., Riordan, P., and Macdonald, D. W. (2012). Does organic farming reduce environmental impacts?–A meta-analysis of European research. *J. Environ. Manage.* 112, 309-320. doi: 10.1016/j.jenvman.2012.08.018

Wang, H. C. (2009). *Urinary purine derivative excretion as a method for estimation of rumen microbial protein production of yak in Qinghai Tibetan plateau* (In Chinese). Ph.D Dissertation. Lanzhou: Lanzhou University.

Wang, H. C., Zhou, J. W., and Long, R. J. (2012). “Comparative study on rumen fermentation characteristics of yak (In Chinese)”. in *Proceedings of the 11th National Symposium on Animal Nutrition*, *Animal Nutrition Branch of China Animal Husbandry and Veterinary Association*. 168.

Wang, W. W. (2020). *Study on characteristics and meachanism of the cross-incubation of rumen inoculum from yak-cattle and Tibetan sheep-Small tailed* (In Chinese). Ph.D Dissertation. Lanzhou: Lanzhou University.

Wang, Y., Li, X., Yang, J., Tian, Z., Sun, Q., Xue, W., et al. (2018). Mitigating greenhouse gas and ammonia emissions from beef cattle feedlot production: a system meta-analysis. *Environ. Sci. Technol.* 52, 11232-11242. doi: 10.1021/acs.est.8b02475

Xue, D., Xiong, W., He, Y. X., Zhu, D., Wu, N., Chen, W., et al. (2017). Respiratory CO2 and CH4 emission fluxes from naturally grazing femTale yak in eastern region of Qinghai-Tibetan Plateau during alpine meadow growing season. *J. Univ. CHIN. Acad. Sci.* 34 : 487-497. doi: 10.7523/j.issn.2095-6134.2017.04.011

Zhang, Z., Xu, D., Wang, L., Hao, J., Wang, J., Zhou, X., et al. (2016). Convergent evolution of rumen microbiomes in high-altitude mammals. *Curr. Biol.* 26, 1873-1879. doi: 10.1016/j.cub.2016.05.012

Zhao, C. C. (2019). *Analysis of physiological and biochemical Indexes and microbial composition of rumen liquid of cattle, Dzo and yak under grazing conditions* (In Chinese). M.S. Dissertation. XianYang: Northwest A＆F University.

Zhou, J. W., Liu, H., Zhong, C. L., Degen, A. A., Yang, G., Zhang, Y., et al. (2018). Apparent digestibility, rumen fermentation, digestive enzymes and urinary purine derivatives in yaks and Qaidam cattle offered forage-concentrate diets differing in nitrogen concentration. *Livest. Sci.* 208, 14-21. doi: 10.1016/j.livsci.2017.11.020
